# Women's dietary diversity is associated with homestead production and market access: A cross‐sectional study in rural Rwanda

**DOI:** 10.1111/mcn.13755

**Published:** 2024-11-03

**Authors:** Theogene Dusingizimana, Gilbert Nduwayezu, Tomas Kjelqvist

**Affiliations:** ^1^ School of Natural Sciences, Technology and Environmental Studies Södertörn University, Alfred Nobel Allé 7 Flemingsberg Huddinge Sweden; ^2^ Department of Food Science and Technology, College of Agriculture, Animal Sciences and Veterinary Medicine University of Rwanda Musanze Rwanda; ^3^ Department of Physical Geography and Ecosystem Science GIS Centre Lund Sweden; ^4^ Department of Civil, Environmental and Geomatics Engineering University of Rwanda Kigali Rwanda

**Keywords:** dietary diversity, homestead production, Rwanda, women

## Abstract

Dietary diversity has been widely used as a proxy indicator for micronutrient adequacy. In low‐ and middle‐income countries (LMICs), including Rwanda, women are at high risk of inadequate micronutrient intake resulting from poorly diversified diets. This study was conducted to examine the factors associated with women's dietary diversity, with emphasis on homestead production diversity and market access in the Northern Province of Rwanda. A cross‐sectional design was used, involving 606 women aged 18–49 years. Linear regression analyses were performed to examine the association between various factors and women's dietary diversity. Results show that 84% of the sample households raised at least one livestock species. Seventy‐one percent of the households had no agricultural land. Eighty percent of those without land had a homestead garden on which they grew food crops, mainly vegetables and fruit trees. The average crop species was 2.3. On average, women consumed 3 out of 9 food groups. The homestead production diversity score was positively associated with women's dietary diversity score (*β* = 0.16, *p* < 0.001). Women's dietary diversity score was negatively associated with distance from the household to the nearest market (*β* = −0.08, *p* = 0.027) and household food insecurity (*β* = −0.06, *p* < 0.001). Maternal education (*p* < 0.001), household wealth index (*p* < 0.05), and ownership of more than 2.5 acres compared to being without land (*p* < 0.05) were associated with women's dietary diversity score. The dietary diversity of women could be enhanced through interventions that promote the diversity of livestock and crop species produced through homestead production. Potential interventions to explore may include integrated farming systems that combine small livestock and crop production utilising improved livestock breeds and high‐quality seeds and planting materials of high‐yielding varieties of fruits and vegetables, along with rainwater harvesting to facilitate small‐scale irrigation. The impact of such interventions on women's dietary diversity can be further reinforced by parallel programmes aimed at improving women's education and the socioeconomic status of households.

## Key messages

- Homestead food production, maternal education, access to land, and household wealth index are positively associated with women's dietary diversity score in the Northern Province of Rwanda.
- Women's dietary diversity score decreases as the distance from the household to the nearest market increases and with higher levels of food insecurity.
- Interventions to promote the diversity of livestock and crop species produced through homestead production can potentially contribute to improving women's access to and consumption of diverse foods. Their impact can be strengthened by complementary programmes that improve women's education, market access, and households' purchasing power.

## Graphical abstract

This study involved 606 women and examined the factors associated with women's dietary diversity score in the Northern Province of Rwanda. It was found that women's dietary diversity score was positively associated with homestead production diversity score, maternal education, household wealth index, and land ownership of more than 2.5 acres. Distance from the household to the nearest market and household food insecurity both were negatively associated with women's dietary diversity.

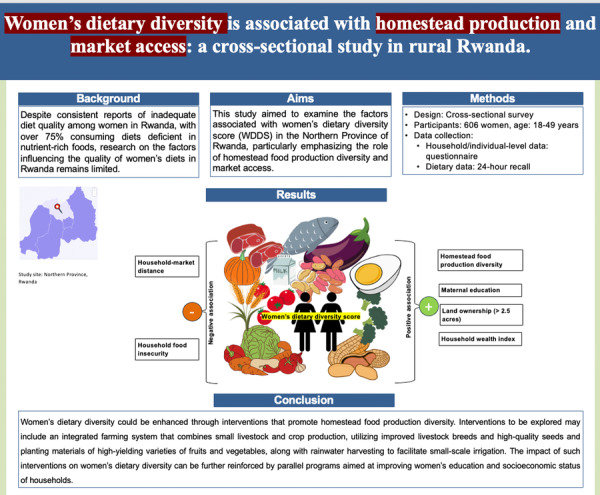

## 1 INTRODUCTION

Despite substantial improvements in the food security situation, more than 820 million people globally lack sufficient food and many more consume poor‐quality diets (Willett et al., 2019). Additionally, more than 2 billion people suffer from hidden hunger (Stevens et al., 2022). Many of the undernourished people are in low‐ and middle‐income countries, mainly in sub‐Saharan Africa and Asia (FAO et al., 2022). National governments in these countries are currently implementing policies to change agricultural production and food systems to address food insecurity and unhealthy diets (FAO et al., 2022), although many of these policies tend to promote a handful of selected high‐yield varieties of crops, mainly wheat, corn, and rice, at the expense of nutrient‐rich crops such as legumes, fruits, and vegetables (IPES‐Food, 2016). However, evidence shows that these high‐yielding varieties have provided limited direct benefits to small‐scale farmers (IFPRI, 2002), who are most likely to be hungry and poor.

Despite the substantial socioeconomic progress that Rwanda has achieved in the past few decades, a large proportion of its population still faces pervasive hunger, food insecurity, and malnutrition. With a Global Hunger Index Score of 25.4, Rwanda is classified as one of the countries with a serious hunger situation (Global Hunger Index, 2023). The most recent Comprehensive Food Security and Vulnerability Analysis (CFSVA) report reveals that in 2021, 20.6% of households in Rwanda were food insecure (NISR, 2022). Additionally, the report indicates that 27% of the households had poor food consumption, characterised by limited consumption of nutrient‐dense foods, such as animal‐source foods, fruits, and vegetables (NISR, 2022). These data are reflected in the high rates of malnutrition, particularly among women. For instance, 17% of women aged 15–49 years in Rwanda suffer from anaemia, with a higher prevalence among pregnant women (24%) (National Institute of Statistics of Rwanda NISR Rwanda, Ministry of Health MOH Rwanda, & ICF, 2021; Ritchie & Roser, 2019). One of the immediate causes of maternal undernutrition, besides disease, is a diet lacking a diversity of foods that are rich in bioavailable micronutrients (Ruel et al., 2010). Recognising this issue, the recent calls to action recommend the government of Rwanda to intensify its efforts to address poor dietary diversity among women (African Union, 2022). Unfortunately, research is limited on factors underlying poor dietary diversity among women. To the best of our knowledge, only one study (Uwase et al., 2024) investigated the factors associated with dietary diversity of women. However, the study was facility‐based and limited to pregnant women, suggesting the need for additional research on this topic.

The agriculture sector in Rwanda serves as a primary source of income and food for rural households. The sector accounts for 28% of the Gross Domestic Product and employs 63% of the population, with over 70% being women (NISR, 2019a). However, the sector is still characterised by small‐scale subsistence farming, with the majority (60%) of households cultivating less than 0.5 of land and 25% having landholding of less than 0.2 ha. This makes many rural smallholder farmers more vulnerable to food insecurity and poor dietary diversity (NISR, 2012).

The current efforts to transform Rwanda's agricultural sector are geared towards changing the sector from a subsistence‐oriented to market‐oriented agriculture. These efforts have primarily focused on, and have indeed contributed to, increased productivity of staple crops, mainly maize, potatoes, rice, beans, and cassava (Nsabimana et al., 2021). However, the impact of these efforts on the quality of diets is still a subject of debate (Dawson et al., 2016) due to their potential negative effects on households' dietary diversity (Del Prete et al., 2019). The need to increase agricultural productivity without compromising household dietary diversity has been recognised (Bizoza, 2021).

Homestead food production has been advocated as a strategy to diversify diets and increase the intake of micronutrients (Gibson, 2011; Shah et al., 2023). Homestead farming systems have long been an integral part of the traditional food production systems in many developing countries (Johnson‐Welch et al., 2000). The primary purpose of homestead farming is to provide food for household consumption, thereby contributing to household food and nutrition security (Guillerme et al., 2011). Evidence suggests that homestead food production is particularly beneficial for vulnerable groups of the population, including pregnant and lactating women (Shetty, 2018). According to Galhena et al. (2013), vulnerable households that have no access to land or income to afford nutritious foods from markets can even benefit more from home gardening. Moreover, studies have shown that the production of fruits and vegetables and small livestock through homestead farming is associated with improved household food security, dietary diversification (Galhena et al., 2013; Rammohan et al., 2019), better maternal diet and nutrition, and women's empowerment (Ruel et al., 2018; Waid et al., 2023). In resource‐poor settings where food insecurity and malnutrition are prevalent, homestead farming is considered a promising strategy for improving diets (Schupp & Sharp, 2012).

In Rwanda, there has been significant attention given to homestead food production as a potential strategy to address high rates of malnutrition. The national nutrition strategy emphasises the importance of homestead food production as one of the key interventions to increase the availability and access to nutrient‐rich foods including fruits, vegetables, and animal‐source foods (Ministry of Health, 2013). As part of this strategy, the establishment of model nutrition gardens at the village level has been proposed to equip people with the necessary skills and information about homestead food production (Ministry of Health, 2013, p. 58).

Despite the recognition of homestead food production, there is limited empirical research examining its impact on dietary diversity, particularly among women in Rwanda. Existing studies have primarily been descriptive case studies that focused on the role of homestead production in improving dietary diversity. However, these studies used a narrative approach and were limited to describing the role of homestead food production in improving food security and dietary diversity in refugee population contexts (Mcoyoo et al., 2016) or in a limited sample (*n* = 42) of households (Sly et al., 2023). Only one peer‐reviewed study by Issahaku et al. (2023) has empirically examined the contribution of homestead gardening on food and nutrition in Rwanda. Although these researchers' findings provided insights into the importance of home gardening, the study focused on outcomes other than women's dietary diversity such as household food insecurity and dietary diversity of children under 5 years of age. The present study complements the work of Issahaku et al. by examining the contribution of homestead food production to the quality of diets of women.

Although the quality of women's diets in Rwanda has consistently been reported as inadequate, with over 75% of women consuming diets lacking in nutrient‐rich foods (NISR, 2019b), there is limited research on factors influencing the quality of women's diets in Rwanda. The aim of this study is to examine the factors associated with the dietary diversity of women in the Northern Province of Rwanda, particularly emphasising the role of homestead food production diversity and market access.

## 2 METHODS

### 2.1 Study settings and context

This study leveraged data from a survey conducted by a Research Programme titled “Undernutrition: An Interdisciplinary Programme focused on children and their mothers” in the Northern Province of Rwanda. The survey aimed to investigate the factors underlying child stunting in the province. In the province, agriculture dominates the economy, but the majority of farmers practice subsistence agriculture. The province has the highest rainfall in Rwanda, making it easier for production. The northern province of Rwanda is part of Rwanda's volcanic region and is considered the breadbasket of the country. However, women's nutrition‐related indicators show a severe situation in the province. For example, in 2021, 11.4% of women in the province suffered from anaemia (haemoglobin <11 g/dL), and only 38% of the women met the minimum dietary diversity (NISR et al., 2021; WFP, 2021). Additionally, 18% of households in the province face food insecurity, marked by limited consumption of nutritionally diverse foods, with a higher incidence among women‐headed households (27%) (NISR, 2022).

### 2.2 Study design, population, and sample size

The aforementioned survey used a cross‐sectional design and a multi‐stage random sampling approach to identify households for inclusion. The main inclusion criteria for the survey were to interview women with children aged 1–3 years. Administratively, the Northern Province is divided into five districts: Burera, Gakenke, Gicumbi, Musanze, and Rulindo. Each district is divided into sectors, which are also divided into cells and villages, with the village being the lowest administrative entity. Specifically, Burera has 17 sectors and 69 cells, Gakenke has 19 sectors and 97 cells, Gicumbi has 21 sectors and 109 cells, Musanze has 15 sectors and 68 cells, and Rulindo has 17 sectors and 71 cells. In the survey, villages were considered as the primary sampling units. The selection of villages, households, and survey participants in the five districts of the province was performed in three steps. The first step involved randomly selecting 134 villages (27 in Burera, 31 in Gakenke, 30 in Gicumbi, 20 in Musanze, and 26 in Rulindo district) from the 2744 existing villages in the five districts of the Northern Province using the geospatial grid system. In the second stage, households were systematically selected from each village using lists of households (sampling frame) obtained from community health workers in charge of maternal and child health at the village level. The number of households selected was proportional to the size of the households in the village. In villages with lower population density, 3–4 households were interviewed, while in villages with higher density, 5–6 households were interviewed. For the systematic sampling, the number of eligible households was divided by the required sample size in each village to obtain the sampling interval (I). The first household was randomly selected between the first and the Ith household in the sampling frame, and subsequent households were identified by adding the interval (I) to the previously selected household. The process was repeated until the required sample size was achieved. In the third stage, a mother in each selected household was interviewed. Mothers were excluded if they were under 18 years old or were severely sick. The sample size of the survey from which the data used for this study were obtained was calculated based on the prevalence of child stunting (40%) in the province, with a desired precision of 4% and a 95% confidence interval. The sample of the main survey consisted of 630 households. However, some participants did not provide complete information, including 21 households (3%) who had no data on farm size. This resulted in 606 of the original samples being considered for the present analysis.

### 2.3 Data collection

Data collection took place between November–December 2021 by trained interviewers. A questionnaire was used to collect data. The questionnaire was first designed in English and translated into the local language, i.e., Kinyarwanda. Then, it was pre‐tested and pre‐programmed on electronic tablets to facilitate data collection, quality checks, and prevent errors associated with paper‐based data collection. The data collected from women included socioeconomic and demographic characteristics, information on household‐level agricultural production and livestock holding, food security, and food consumption using a 24‐h recall method, which allowed the collection of data on all food items consumed by the women in the past 24 h before the survey.

### 2.4 Primary outcome of interest

The dietary diversity score of women (WDDS) was the primary outcome measure for this analysis. The WDDS was used as a continuous variable. Dietary diversity has been used as one of the measures of quality of human diets (Arimond et al., 2010). A 24‐h food recall methodology was used to collect information about foods consumed by the women within the last 24 h of the survey. The foods were aggregated into nine food groups in accordance with the previous methodology (Arimond et al., 2010). The food groups considered were (1) cereals, white roots, and tubers; (2) legumes, nuts, and seeds; (3) vitamin A‐rich green leafy vegetables; (4) other vitamin A‐rich fruits and vegetables; (5) dairy; (6) meat, poultry, and fish; (7) eggs; (8) other fruits; and (9) other vegetables. The consumption of a food item from any food group was coded as one and zero otherwise. We used the nine food groups because of an oversight during data collection, which resulted in the non‐separation of pulses, nuts, and seeds.

### 2.5 Exposures of interest

The two exposures of interest in the present study were homestead food production diversity and access to markets. Questions about homestead food production were incorporated into the questionnaire of the main survey questions about agricultural production. These questions sought to elicit information about crop and livestock food production. Specifically, respondents were asked: (i) if they owned a homestead garden (small home adjunct plots primarily for vegetable production) around their houses upon which they food crops, fruits, or vegetables; (ii) for those who owned homestead gardens, which types of crop/fruit/vegetables were grown at the time of the survey; (iii) if they owned livestock; (iv) which livestock species they raised in or around their homes; and (v) if they owned another agricultural land used for crop production. Home production diversity was measured as a simple count of all crops/fruits/vegetables and livestock produced in the homestead garden. Crops/fruits/vegetables grown in the homestead garden of sample households included maize, yam, beans, peas, banana (yellow or green), sweet potatoes, Irish potatoes, pumpkin, dodo (i.e., amaranths), carrots, cabbage, tomatoes, onions, eggplants, avocado, orange, lemon, passion fruits, and papaya. Livestock raised included cattle, pigs, goats, sheep, poultry, and rabbits. A value of 1 was assigned to each crop/fruit/vegetable species or livestock species produced. The sum of the counts was used as an indicator of homestead production diversity. To ensure the accuracy of the data, interviewers also requested the respondents permission to observe the crops/fruits/vegetables and livestock.

Distance from the household to the nearest market was used as a proxy for market access. In the present study, Global Positioning System coordinates for the location of households were recorded during data collection. These coordinates were used to determine the distance from households to the nearest market. The distance (crow fly) was calculated using ArcGIS Pro software (version 3.2). In the absence of road networks, we employed the crow fly distance with the assumption that households purchase foods or sell the surplus of what they produce from the nearest markets.

### 2.6 Other variables

Other variables representing individual and household demographic and socioeconomic characteristics were also considered in our analysis. Individual characteristics included age, years of education, and marital status of the respondents. Household characteristics included the gender of the household head, the number of household members, land size, household food insecurity, and household wealth index. We used a principal component analysis to derive household relative household wealth index (Vyas & Kumaranayake, 2006). Twelve variables were used for PCA, including access to electricity, ownership of durable assets (bicycle, mobile phone, radio, television, steam iron, mattress), the quality of house walls, ownership of a kitchen that is separate from the main house, ownership of health insurance for all household members, access to improved water source, and ownership of improved toilet facility. The score of the first Principal Component was used to categorise households into tertiles, representing wealth categories. Household food insecurity was determined using the Household Food Insecurity Access Score questionnaire (Ballard et al., 2011). This was a continuous variable ranging between 0 and 27, meaning that the higher the insecure food access score, the more food insecure the household.

### 2.7 Data analysis

Descriptive statistics were performed to summarise the sample characteristics. Continuous variables were summarised using means and standard deviations and categorical variables using frequencies and percentages. Linear regression analysis was performed to examine the associations between WDDS and homestead production diversity as well as other factors. All assumptions were assessed. Multicollinearity was checked using the variance inflation factor (VIF). The VIF values of all predictors were <5, indicating that multicollinearity was not a concern (Gareth et al., 2013). Homoscedasticity was assessed by visual inspection of a plot of standardised residuals *v*. standardised predicted values. All statistical tests were performed at a 5% level of significance. Data were analysed using Stata version 18 (StataCorp, 2023).

### 2.8 Ethical consideration

The data used for this analysis was collected as part of a larger study that was conducted according to the guidelines laid down in the Declaration of Helsinki. The study received ethical clearance from the National Health Research Committee and the appropriate Institutional Review Boards. District offices and community leaders were also informed and assisted with access to the community. Informed verbal consent was obtained from each respondent before data collection.

## 3 RESULTS

### 3.1 Descriptive statistics

Descriptive statistics describing the household characteristics are presented in Tables 1 and 2. The average household size was approximately five people. More than half (57%) had no formal education, of which 25% had no education at all and 32% did not complete primary education. On average, people travelled 3 km to reach the nearest market. Eighty‐four percent (84%, *n* = 511) of the sample households raised at least one livestock species (mean livestock diversity score = 1.4). Among these, about two‐thirds of the households (65%, *n* = 395) raised cattle, followed by poultry (21%), and 20% both goats and pigs. Seventy‐one percent (71%, *n* = 418) of the households had no agricultural land. Interestingly, 80% (*n* = 335; data not presented) of those without land had a homestead garden on which they grow food crops, mostly vegetables, and fruit trees. The average crop species was 2.3.

**Table 1:** Sample characteristics.

Variables	*n*	Mean (SD) or mean proportions
Homestead production diversity		
Homestead production diversity score	605	3.9 (2.3)
Crop species diversity	605	2.3 (1.7)
Livestock species diversity	605	1.4 (1.0)
Household characteristics		
Maternal age (years)	606	31.6 (7.2)
Gender of household head (male)	606	0.91
Marital status (married)	534	0.88
Maternal education (years)	605	5.5 (3.1)
Maternal education		
None or incomplete primary education	157	0.57
Completed primary education	156	0.25
Some secondary or technical education	101	0.16
Tertiary education	3	0.50
Household wealth index category		
Lowest tertile	205	0.34
Middle tertile	200	0.33
Upper tertile	201	0.33
Household size	605	5.2 (2.0)
Household ownership of durable assets		
Mobile phone	517	0.85
Radio	209	0.34
TV	23	0.04
Bicycle	71	0.12
Iron	47	0.08
Mattress	467	0.77
Household has access to improved drinking water source	382	0.63
Household has access to electricity	228	0.38
Household ownership of health insurance	583	0.96
Household has access to improved toilet facility (yes)	373	0.45
Homestead garden ownership (yes)	506	0.84
Agricultural land ownership (*n* = 585)		
No land landless	418	0.71
<2.5 acres (<1 ha)	125	0.21
≥2.5 acres (>1 ha)	42	0.07
Household raises some livestock (yes)	511	0.84
Livestock species raised		
Cattle	395	0.65
Pigs	114	0.18
Goat	120	0.19
Sheep	91	0.15
Rabbit	35	0.5
Poultry	128	0.21
Household food insecurity score (count)	605	10.1 (7.2)
Distance to the nearest market (km)	605	3.0 (1.6)

**Table 2:** Food groups consumed and dietary diversity of women (*n* = 606).

Variable	*n*	Mean (SD) or %
% of women who consumed different food groups		
Grains, white roots, and tubers	596	93.3
Legumes and nuts	536	88.0
Milk and milk products	114	18.7
Meat, poultry, and fish	84	13.8
Eggs	22	3.6
Vitamin A‐rich dark green leafy vegetables	399	65.5
Other vitamin A‐rich fruits and vegetables	184	30.2
Other vegetables	277	45.5
Other fruits	46	7.6
Average dietary diversity score	605	3.42 (1.4)

Table 2 shows the percentage of women who consumed different food groups. On average, women consumed 3 out of 9 food groups. The starchy staple food group was the highest to be consumed (93%, *n* = 596), followed by legumes and nuts (88%, *n* = 536), and vitamin A‐rich dark green vegetables (65%). Animal‐source foods were the least consumed food groups (19%, 14%, and 3% for dairy, flesh meat and fish, and eggs, respectively).

### 3.2 Women's dietary diversity and associated factors

Table 3 shows the regression results of factors associated with WDDS. The homestead production diversity score had a positive and significant association with WDDS. Every increase in the homestead production diversity score was associated with a 0.17 unit increase in WDDS. We also observed a statistically significant and positive association between land ownership and WDDS. On average, the WDDS in households owning more than 2.5 acres was 0.43 higher than that of women in households without land. There was also a positive and significant association between WDDS maternal education, household wealth index, marital status, and WDDS. On average, for each additional year of maternal education, we observe an increase of 0.09 units in WDDS. While the *p*‐value associated with marital status was less than 5%, suggesting statistical significance, the confidence interval for this variable crosses 1. This suggests some level of uncertainty in the precise estimate of the effect size. There was also a statistically significant and negative association between distance from the household to the nearest market and food insecurity score and WDDS. Every increase in the 1 km distance from households to the nearest market was associated with a 0.09 unit reduction in WDDS. Similarly, every increase in 1 score in household food insecurity was associated with 0.06 units of reduction in WDDS.

**Table 3:** Factors associated with women's dietary diversity.

Variables	*β*	95% CI	*p*‐Value
Homestead production diversity score	0.16	0.098, 0.226	<0.001
Distance to the nearest market (km)	−0.08	−0.147, −0.009	0.027
Maternal education (years)	0.09	0.047, 0.130	<0.001
Maternal age (years)	0.02	−0.009, 0.049	0.192
Gender of household head (male)	−0.34	−0.924, 0.189	0.195
Age of partner (years)	−0.00	−0.023, 0.015	0.678
Education of partner (years)	0.01	−0.023, 0.047	0.514
Marital status (married)	0.67	0.10, 1.250	0.020
Household size	−0.06	−0.132, 0.033	0.130
Agricultural land size (ref. = landless)			
<2.5 acres	−0.08	−0.504, 0.348	0.719
≥2.5 acres	0.46	−0.166, 0.745	0.002
Household food insecurity score	−0.06	−0.073, −0.037	<0.001
Household has a homestead garden (yes)	−0.13	−0.482, 0.221	0.467
Household wealth index (ref.: lowest tertile)			
Middle tertile	0.28	0.004, 0.574	0.047
Upper tertile	0.43	0.127, 0.728	0.005
No. observations	543		
Adjusted *R* ^2^	0.30		

*Note*: *β*, standardised coefficients; 95% CI, 95% confidence interval.

## 4 DISCUSSION

The present study has found a positive association between homestead food production diversity and WDDS. While research on factors influencing WDD in Rwanda is limited, several intervention studies demonstrated that homestead food production can increase WDD. In Bangladesh, Waid et al. (2023) found that homestead food production intervention increased dietary diversity in women and that the impacts persisted even after the end of the intervention. Similar findings were documented in Nepal, where Dulal et al. found positive associations between participation in homestead food production activities and maternal and child dietary diversity, after controlling for socioeconomic and demographic confounders that could potentially influence dietary diversity. However, the associations were statistically significant in the winter season and not in the rainy season (Dulal et al., 2017). A 16‐week intervention study conducted in one of the Burera districts of the Northern Province of Rwanda (Burera) (Sly et al., 2023) reported that participation in kitchen gardening intervention was associated with an increased household dietary diversity. In the context of the present study setting where access to agricultural land appears to be limited, our findings demonstrate that homestead food production can play a significant role in improving women's access to and consumption of a variety of foods.

The negative association between household food insecurity and WDDS observed in the present study was not surprising. Previous research has found an inverse relationship between food insecurity and dietary diversity (Hashmi et al., 2021). In Rwanda, household food insecurity was also found to be associated with the consumption of monotonous diets, an indicator of low dietary diversity among households (NISR, 2012). In other words, women from food insecure households are more likely to consume limited diverse foods. This finding underscores the need to strengthen programmes to enhance household food security.

The positive association between maternal education and women's dietary diversity observed in the present study has also been found in other studies (Makate & Nyamuranga, 2023). Literature shows that educated women are more likely to have better nutrition knowledge than uneducated mothers, which may translate into improved dietary diversity (Makate & Makate, 2018). Education is also an important component of women's empowerment, which enhances women's ability to take control over their own health, including their diets (Kassie et al., 2020). However, education on its own is not sufficient, particularly in contexts where households have no access to food (Muehlhoff et al., 2017; Ruel & Alderman, 2013). Thus, given the negative association between food insecurity and WDDS observed in the present study, our findings confirm findings from other studies suggesting that WDDS would be improved if programmes to provide educational opportunities to women are coupled with measures to reduce households' access to food through homestead food production.

Our findings of a negative and statistically significant association between market access and WDDS are in line with previous research from other African countries (Usman & Callo‐Concha, 2021; Usman & Haile, 2022). Our findings are also in agreement with Sibhatu et. al.'s findings, which indicated that households in remote regions had lower dietary diversity in the context of Asian and African countries (Sibhatu et al., 2015). In Rwanda, households purchase two‐thirds of their food from markets and spend more than 65% of their household budget on food. Additionally, in many villages without markets, it takes about 2 h to reach the nearest market. Therefore, our findings suggest that improving market access may be a promising strategy for households and women in particular to increase their dietary diversity. However, our observation of a statistically significant association between the wealth index and WDDS aligns with previous literature, which shows that women's diet quality follows a socioeconomic gradient, with women from higher socioeconomic positions consuming more diverse foods compared to those from lower socioeconomic positions (McLeod et al., 2011). This suggests that interventions aimed at improving market access should also focus on enhancing households' purchasing power to ensure that they have both physical and economic access to markets. As expected, access to more land, i.e., ≥2.5 acres, was significant and positively associated with WDDS. Many studies elsewhere have found a positive association between women's dietary diversity and household farm production diversity. Unfortunately, the influence of household agricultural production diversity on WDDS was not examined as this was beyond the scope of the present study.

### 4.1 Policy and practical implications of findings

Findings from the current study have several implications for policies, programmes, and initiatives aimed to improve the quality of foods consumed in households in Rwanda, especially by women. For example, with the current Land Use Consolidation policy, where farmers are mobilised/required to consolidate their lands to grow only one crop (depending on location) as a requirement to benefit from government agricultural subsidies (e.g., improved seeds and other inputs), many scholars (Bizoza, 2021; Nsabimana et al., 2021) have raised questions as to how the needs for households to consume a varied and balanced diet will be met. Thus, our findings indicated that homestead food production may be considered as part of the strategies to meet the demand for households to produce and consume diversified foods. In addition, within the context of our study settings, where 70% of households have no agricultural land, of which 80% appear to depend on homestead gardens to grow foods, our findings also suggest that homestead production can potentially contribute to improving women's access to and consumption of a variety of foods. Moreover, given that agriculture in Rwanda is highly dependent on rainfalls, which limits the production of a variety of crops (Weatherspoon et al., 2021), homestead food production may be an appealing alternative for households to diversify their foods. However, it is important to highlight that several factors need to be considered if households tap into the benefits of homestead farming. For example, households need to be supported with simple farming technologies such as small‐scale irrigation technologies to increase food production or food processing technologies to preserve the quality of foods, especially vegetables that are highly perishable.

### 4.2 Limitations

The present study has some limitations. Firstly, its cross‐sectional nature means that causality cannot be established. Secondly, although the sample was fairly representative of the population in the northern province, the findings may not be fully generalisable because the survey was conducted during the short rainy season. Seasonality is known to influence agricultural production and food consumption. Thirdly, we used the WDDS, which is based on 9 food groups, instead of Minimum Dietary Diversity for Women (MDD‐W), which is a 10‐food group indicator. This has several drawbacks as it does not allow us to distinguish between women with low or adequate dietary diversity. Future research should use the most recent MDD‐W, which allows this classification. Fourthly, recall bias and social desirability may have also influenced our results. To minimise social desirability bias, data collectors informed respondents about the study objectives. Recall bias was also minimised by specifying the time of food consumption and by probing to get specific information. Lastly, unknown and unmeasured confounding factors may also affect the results. However, despite these limitations, the study used a large and representative sample, with high‐quality control measures during data collection. Fourth, while the present study emphasised on homestead food production diversity, the relationships between this variable could be influenced by production diversity on other plots of land cultivated by the households. Many studies elsewhere have found a positive association between women's dietary diversity and household farm production diversity. Unfortunately, the influence of household agricultural production diversity on WDDS was beyond the scope of the present study. Future research considering the diversity of both homestead food production and production on other plots of land is recommended and would provide additional insights.

## AUTHOR CONTRIBUTIONS

**Theogene Dusingizimana**: Conceptualisation; writing original draft; formal analysis. **Gilbert Nduwayezu**: Data curation; review; editing. **Tomas Kjelqvist**: Conceptualisation; review; editing.

## CONFLICT OF INTEREST STATEMENT

The authors declare no conflicts of interest.

## Data Availability

The data that support the findings of this study are available on request from the corresponding author. The data are not publicly available due to privacy or ethical restrictions.
